# Association between Pre-Admission Polypharmacy and Functional Status at Discharge in Patients with Acute Stroke: A Retrospective Observational Study

**DOI:** 10.31662/jmaj.2025-0597

**Published:** 2026-07-10

**Authors:** Hiroyasu Murata

**Affiliations:** 1 Department of Physical Therapy, Faculty of Health Science, Kyorin University, Mitaka, Tokyo, Japan

**Keywords:** stroke, polypharmacy, Functional Independence Measure, activities of daily living, rehabilitation

## Abstract

**Introduction:**

Polypharmacy is prevalent among stroke patients and has been associated with adverse outcomes in elderly individuals. However, its impact on functional recovery in the acute phase of stroke remains uncertain. The present study examined the association between polypharmacy at admission and functional outcomes at discharge in patients with acute stroke.

**Methods:**

A retrospective observational study was conducted on patients aged ≥18 years who were admitted with ischemic or hemorrhagic stroke to a Japanese university hospital between September 2022 and October 2023. Polypharmacy was defined as the use of ≥5 pre-admission regular medications confirmed at the time of admission; medications newly initiated after admission were not included. The primary outcome was the total Functional Independence Measure (FIM) score at discharge, and FIM gain during hospitalization was a secondary outcome. Patients were divided into two groups: those who were prescribed multiple medications (polypharmacy group) and those who were not (non-polypharmacy group). Multiple linear regression analysis was used to evaluate whether polypharmacy was independently associated with FIM at discharge after adjustment for age, comorbidities, pre-stroke disability, stroke severity, serum albumin, and baseline FIM scores. A sensitivity analysis was conducted in patients aged ≥65 years.

**Results:**

Of the 339 eligible patients, 303 were included in the final analysis; 170 (56.1%) had polypharmacy. Patients with polypharmacy exhibited lower total FIM scores at admission and discharge, in addition to diminished FIM gain and prolonged hospital stays when compared with patients without polypharmacy. In the fully-adjusted model, polypharmacy remained independently associated with lower total FIM at discharge (adjusted difference −5.3 points, 95% confidence interval −9.5 to −1.1; p = 0.013). Analogous associations were observed in the analysis restricted to patients aged ≥65 years.

**Conclusions:**

Pre-admission polypharmacy was associated with lower functional status at discharge, but not with FIM gain, in patients with acute stroke. These findings suggest that pre-admission polypharmacy may serve as a marker of clinical complexity and help identify patients at risk of poorer discharge functional status.

## Introduction

Stroke is a leading cause of death and disability worldwide. Post-stroke functional recovery is therefore crucial for restoring independence, and for reducing the burden on caregivers and healthcare systems. The acute and subacute phases, in particular, represent a critical window during which functional recovery is most pronounced, making rehabilitation a major priority in stroke care. Against this backdrop, the potential impact of polypharmacy on stroke rehabilitation has recently attracted growing attention ^(1), (2)^.

Polypharmacy is commonly defined as the use of ≥5 medications concomitantly ^(3)^. Although this definition has been widely used, it has primarily been established in studies of older adults. However, the term does not simply refer to the use of a large number of prescribed drugs; rather, it refers to a state in which medication burden is associated with an increased risk of adverse drug events, medication errors, and poor adherence. Previous studies have indeed shown that polypharmacy is associated with frailty ^(4)^, falls ^(5)^, hospital readmission ^(6)^ and higher mortality ^(7), (8)^.

A multitude of mechanisms through which polypharmacy may impede post-stroke functional recovery have been postulated. In patients with subacute stroke, polypharmacy has been reported to be negatively associated with Activities of Daily Living (ADL) scores, energy intake, and the likelihood of discharge home ^(1), (2), (9)^. Conversely, deprescribing interventions have been linked to improved functional recovery and higher home discharge rates ^(10)^. However, it should be noted that the aforementioned studies were conducted in subacute stroke rehabilitation settings, and the evidence in patients with acute stroke remains limited. In the acute phase, the presence of polypharmacy at admission has been associated with increased stroke severity in patients with ischemic stroke ^(11)^ and with reduced odds of a favorable outcome after alteplase treatment ^(12)^. However, the association between polypharmacy and ADL-related outcomes in patients with acute stroke remains to be fully elucidated.

This distinction is clinically important because, in acute care hospitals, early identification of patients at risk of poor discharge functional status may help guide rehabilitation planning and discharge preparation from the early stage of hospitalization. The acute stroke setting also differs from convalescent rehabilitation settings because treatment decisions, rehabilitation initiation, and discharge planning must be made within a much shorter time frame. Therefore, this study aimed to examine the association between pre-admission polypharmacy identified at hospital admission and functional outcomes in patients with acute stroke. The primary outcome was total Functional Independence Measure (FIM) score at discharge, and the secondary outcome was FIM gain during hospitalization.

## Materials and Methods

### Participants and setting

This single-center retrospective observational study included consecutive patients admitted to a Japanese university hospital between September 2022 and October 2023. Eligible patients were identified using the hospital’s electronic medical records. This study was reported in accordance with the Strengthening the Reporting of Observational Studies in Epidemiology (STROBE) statement. The study protocol was approved by the Ethics Committee of Kyorin University School of Medicine (approval no. R04-045). The requirement for informed consent was waived because of the retrospective observational design. Instead, an opt-out consent procedure was implemented by disclosing study information on the institutional website and allowing patients to refuse participation.

### Eligibility

Patients aged ≥18 years who were admitted with ischemic or hemorrhagic stroke were eligible for inclusion. Patients were excluded if they had a pre-stroke modified Rankin Scale (mRS) score of 5, because functional recovery during hospitalization could not be meaningfully evaluated in patients with severe pre-existing disability. Patients who died during hospitalization were also excluded because discharge FIM and FIM gain could not be meaningfully assessed in these patients. Patients with missing data for variables required in the multivariable analyses were excluded using complete-case analysis.

### Data collection

Data on age, sex, body mass index (BMI), stroke type, acute stroke treatment, comorbidities, serum albumin, pre-stroke functional status, stroke severity, and ADL were collected from electronic medical records. Stroke type was categorized as ischemic or hemorrhagic stroke based on the diagnosis recorded in the electronic medical records. The severity of comorbidities was assessed using the Charlson Comorbidity Index (CCI) ^(13)^, with higher scores indicating a greater burden of comorbidity. Stroke severity at admission was assessed using the National Institutes of Health Stroke Scale (NIHSS) ^(14)^, which ranges from 0 to 42, with higher scores indicating more severe neurological impairment. Pre-stroke functional status was assessed by physicians using the mRS ^(15)^, which ranges from 0 to 6, with higher scores indicating greater disability. Data on acute stroke treatments, including thrombolysis, mechanical thrombectomy, and hematoma evacuation, were also collected. Serum albumin was measured from a blood sample obtained at admission. ADL was assessed using the FIM ^(16)^, an 18-item instrument that evaluates independence in ADL, with total scores ranging from 18 to 126; higher scores indicate greater independence. FIM was assessed by therapists who had undergone standardized in-hospital training in FIM scoring. Admission FIM was evaluated, in principle, within 72 hours after hospitalization, and discharge FIM reflected the patient’s functional status up to the day before discharge. When scoring disagreements arose, they were resolved through interdisciplinary discussion among physical therapists, occupational therapists, and speech-language pathologists.

### Polypharmacy

Polypharmacy was defined as the use of ≥5 regular pre-admission medications at the time of hospital admission ^(3)^. As part of routine clinical practice, ward pharmacists performed medication reconciliation using brought-in medications, medication notebooks, and information obtained from patients and/or their family members. Medications newly initiated after admission were excluded, and only pre-admission regular medications were counted. As-needed medications and topical agents, including eye drops, patches, and inhalers, were not counted.

In addition to the total number of pre-admission medications, major pre-admission medication classes were recorded. These included antithrombotic agents, antihypertensive agents, lipid-lowering agents, antidiabetic agents, gastric acid suppressants, hypnotics, anxiolytics, antidepressants, antipsychotics, analgesics, antiepileptic drugs, and strong anticholinergic drugs. Psychotropic drug use was defined as the use of hypnotics, anxiolytics, antidepressants, or antipsychotics. Analgesics were recorded as a separate medication class and were not included in psychotropic drugs.

Strong anticholinergic drug use was defined as the use of at least one medication with an Anticholinergic Cognitive Burden (ACB) score of 3 ^(17)^. We selected the ACB score because it is a widely used and clinically interpretable scale for classifying medications according to their potential cognitive anticholinergic burden ^(17), (18)^. Given the focus of the present study on ADL-related outcomes after stroke, for which cognition, attention, and alertness may be clinically relevant, the ACB score was considered suitable for the supplementary description of strong anticholinergic drug use in this cohort.

### Outcomes

The primary outcome was total FIM score at discharge, which reflected ADL status at discharge from the acute care hospital. The secondary outcome was FIM gain during hospitalization, defined as discharge total FIM score minus admission total FIM score.

### Sample size calculation

The sample size calculation was based on previous studies. The detectable between-group difference was set at 22 points, corresponding to the minimal clinically important difference (MCID) for total FIM reported in a previous study of acute stroke ^(19)^. The standard deviation was assumed to be 40 based on historical data from our hospital. The prevalence of polypharmacy was assumed to be approximately 30%, with an allocation ratio of 1:2. With 90% power and a two-sided alpha level of 0.05, the required sample sizes were estimated to be 56 and 106 patients in the polypharmacy and non-polypharmacy groups, respectively.

### Statistical analysis

Missing data were handled by complete-case analysis. Patients with missing data for any variables included in the primary multivariable model were excluded from the final analysis. The number of excluded patients and reasons for exclusion are shown in Figure 1.

**Figure 1.**
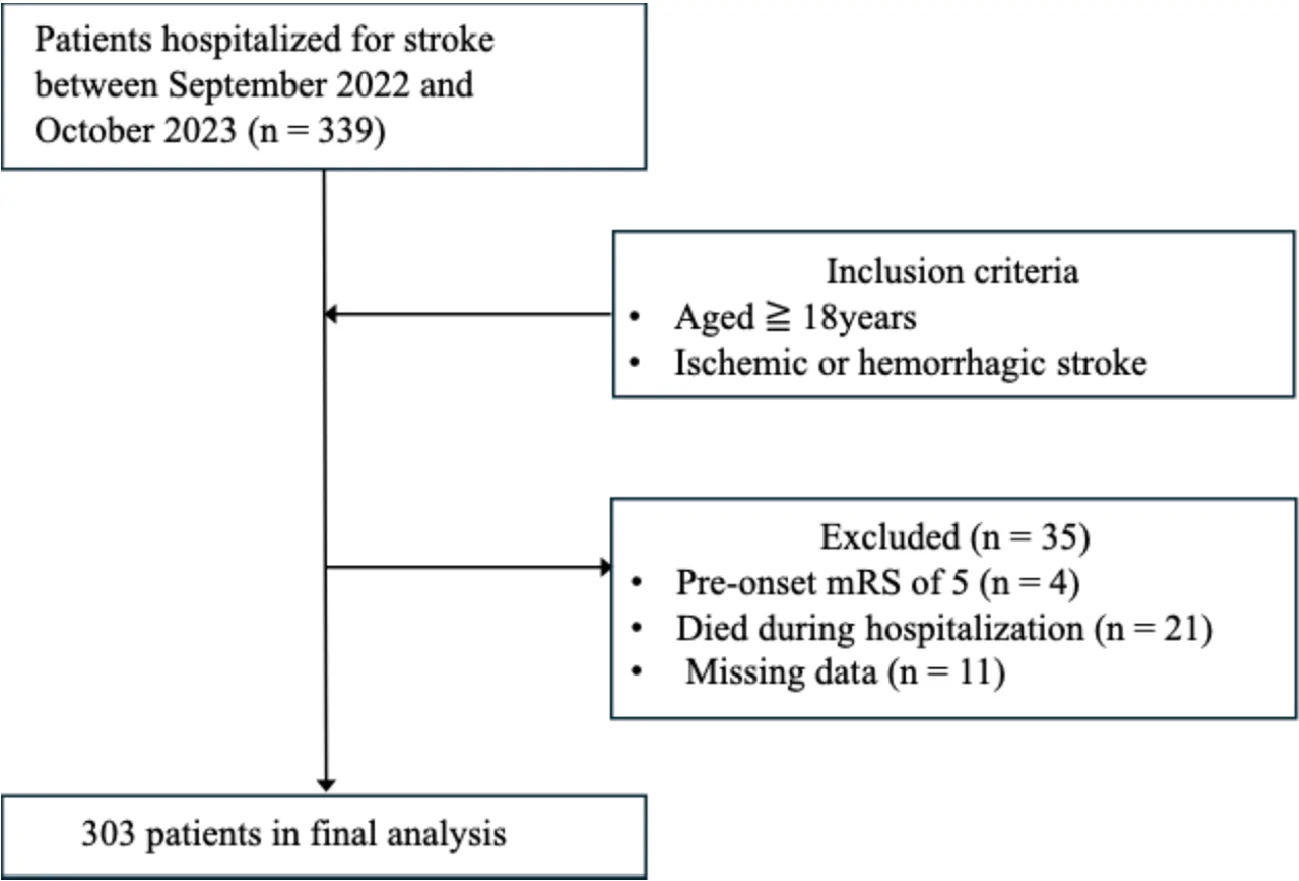
Flowchart of participant screening and inclusion criteria. A total of 339 patients with acute ischemic or hemorrhagic stroke admitted between September 2022 and October 2023 were screened. Patients with pre-stroke modified Rankin Scale (mRS) score of 5 (n = 4), in-hospital death (n = 21), and missing data (n = 11) were excluded, leaving 303 patients for the final analysis.

Continuous variables are presented as medians and interquartile ranges (IQRs), and categorical variables are presented as numbers and percentages. Between-group comparisons according to pre-admission polypharmacy status were performed using the Mann-Whitney U test for continuous variables and the chi-square test or Fisher’s exact test, as appropriate, for categorical variables.

Multiple linear regression analysis was used to examine the association between pre-admission polypharmacy and total FIM score at discharge. In the primary analysis, total FIM score at discharge was used as the dependent variable. Pre-admission polypharmacy and the following covariates were simultaneously entered into the model using the forced-entry method: age, sex, BMI, CCI, stroke type, pre-stroke mRS, NIHSS at admission, serum albumin at admission, admission motor FIM score, and admission cognitive FIM score. Pre-stroke mRS was dichotomized as 0-2 and 3-4.

The covariates were selected a priori based on clinical relevance and previous evidence regarding predictors of post-stroke functional outcome. Specifically, age and sex were included as demographic factors; BMI, CCI, and serum albumin were included to account for body composition, comorbidity burden, and systemic inflammatory status or overall clinical condition; stroke type and NIHSS at admission were included to account for stroke characteristics and neurological severity; pre-stroke mRS was included to account for premorbid disability; and admission motor and cognitive FIM scores were included to account for baseline functional status.

Length of hospital stay was not included as a covariate in the primary model because it may lie on the causal pathway between medication burden and discharge functional status; adjustment for length of stay could therefore result in overadjustment. Multicollinearity was evaluated using the variance inflation factor (VIF), with a VIF value <10 considered to indicate the absence of serious multicollinearity. Linear regression assumptions were assessed by visual inspection of residual plots and normal Q–Q plots. Homoscedasticity was examined using residual-versus-fitted plots.

FIM gain was analyzed as the secondary outcome using multivariable linear regression with the same covariate set as the primary model. Several sensitivity and supplementary analyses were also conducted. First, heteroscedasticity-consistent robust standard errors using the HC3 estimator were calculated for the primary model to examine the robustness of the association between pre-admission polypharmacy and discharge total FIM. Second, additional models adjusted for length of hospital stay were performed for discharge total FIM and FIM gain to examine whether the findings remained consistent after accounting for differences in hospitalization duration. Third, a sensitivity analysis restricted to patients aged ≥65 years was performed using the same covariate set as in the primary analysis. Fourth, exploratory age-stratified analyses were conducted in three age groups: 18-64, 65-74, and ≥75 years. Because the sample size decreased after stratification, these analyses were performed using a parsimonious model including pre-admission polypharmacy, age, pre-stroke mRS, NIHSS at admission, admission motor FIM score, and admission cognitive FIM score. The interaction between pre-admission polypharmacy and age group was also formally tested.

Additional exploratory analyses were performed to examine medication-related factors in more detail. An additional model adjusted for psychotropic drug use was performed because psychotropic medications may influence attention, alertness, balance, and participation in rehabilitation, which could affect ADL-related outcomes after stroke. Finally, the number of pre-admission medications was analyzed as a continuous variable instead of binary polypharmacy using the same covariate set as in the primary model.

All statistical analyses were conducted using R software, version 4.2.3 (R Foundation for Statistical Computing, Vienna, Austria). Statistical significance was set at p < 0.05.

## Results

A total of 339 patients with stroke were admitted during the study period. After excluding patients with a pre-stroke mRS score of 5 (n = 4), patients who died during hospitalization (n = 21), and patients with missing data (n = 11), the final analysis included 303 patients (Figure 1).

Table 1 shows the baseline characteristics and in-hospital outcomes according to pre-admission polypharmacy status. The median age was 77 years (IQR, 66-85 years), and 161 patients (53.1%) were male. Pre-admission polypharmacy was observed in 170 patients (56.1%). Compared with patients without polypharmacy, those with polypharmacy were significantly older and had higher CCI scores and worse premorbid functional status as assessed by pre-stroke mRS (all p < 0.001). Stroke type also differed significantly between the groups (p = 0.030), and serum albumin levels at admission were lower in the polypharmacy group (4.1 [3.8-4.4] vs. 3.9 [3.5-4.3] g/dL; p = 0.006). There were no statistically significant differences between the groups in NIHSS at admission or the use of thrombolysis, mechanical thrombectomy, or hematoma evacuation.

**Table 1.** Baseline Characteristics and In-Hospital Outcomes According to Pre-Admission Polypharmacy Status.

Variable	Overall (n = 303)	Non-polypharmacy (n = 133)	Polypharmacy (n = 170)	p Value
Age, years	77 [66-85]	70 [58-79]	81 [73-87]	<0.001
Sex, male, n (%)	161 (53.1)	72 (54.1)	89 (52.4)	0.847
BMI, kg/m^2^	22.0 [19.4-24.9]	22.5 [19.9-25.4]	21.8 [19.2-24.4]	0.136
Charlson comorbidity index	1 [0-2]	0 [0-1]	2 [1-3]	<0.001
Stroke type, n (%)				0.030
Ischemic stroke	227 (74.9)	91 (68.4)	136 (80.0)	
Hemorrhagic stroke	76 (25.1)	42 (31.6)	34 (20.0)	
Pre-stroke mRS, n (%)				<0.001
0	182 (60.1)	103 (77.4)	79 (46.5)	
1	31 (10.2)	7 (5.3)	23 (13.5)	
2	32 (10.6)	9 (6.8)	23 (13.5)	
3	35 (11.6)	11 (8.3)	24 (14.1)	
4	24 (7.9)	3 (2.3)	21 (12.4)	
NIHSS at admission	5 [2-14]	4 [2-14]	5 [2-14]	0.576
Serum albumin at admission, g/dL	4.0 [3.6-4.3]	4.1 [3.8-4.4]	3.9 [3.5-4.3]	0.006
Thrombolysis, n (%)	24 (7.9)	12 (9.0)	12 (7.1)	0.679
Mechanical thrombectomy, n (%)	19 (6.3)	9 (6.8)	10 (5.9)	0.939
Hematoma evacuation, n (%)	2 (0.7)	0 (0.0)	2 (1.2)	0.506
FIM at admission				
Motor	33 [13-56]	39 [17-58]	32 [13-53]	0.051
Cognitive	27 [13-33]	29 [15-34]	25 [12-33]	0.061
Total	58 [32-87]	68 [34-91]	56 [29-82]	0.048
mRS at discharge, n (%)				0.170
0	47 (15.5)	27 (20.3)	20 (11.8)	
1	52 (17.2)	26 (19.5)	26 (15.3)	
2	41 (13.5)	19 (14.3)	22 (12.9)	
3	49 (16.2)	18 (13.5)	31 (18.2)	
4	69 (22.8)	28 (21.1)	41 (24.1)	
5	45 (14.9)	15 (11.3)	30 (17.6)	
NIHSS at discharge	2 [0-6]	2 [0-4]	2 [0-8]	0.092
FIM at discharge				
Motor	67 [30-86]	76 [42-90]	58 [22-80]	<0.001
Cognitive	30 [16-35]	32 [21-35]	27 [14-34]	0.010
Total	94 [51-120]	107 [67-124]	80 [39-113]	<0.001
FIM gain	22 [8-35]	27 [16-37]	18 [4-32]	<0.001
Length of hospital stay, days	19 [13-36]	18 [12-29]	20 [14-41]	0.007
Discharge destination				0.084
Home, n (%)	151 (49.8)	72 (54.1)	79 (46.5)	
Rehabilitation hospital, n (%)	104 (34.3)	48 (36.1)	56 (32.9)	
Long-term care hospital, n (%)	35 (11.6)	10 (7.5)	25 (14.7)	
Others, n (%)	13 (4.3)	3 (2.3)	10 (5.9)	

Continuous variables are presented as medians [interquartile range], and categorical variables are presented as numbers (%). p Values were calculated using the Mann-Whitney U test for continuous variables and the chi-square test or Fisher’s exact test, as appropriate, for categorical variables. FIM gain was defined as discharge total FIM score minus admission total FIM score. Pre-admission polypharmacy was defined as the use of five or more regular pre-admission medications. BMI: body mass index; FIM: Functional Independence Measure; mRS: modified Rankin Scale; NIHSS: National Institutes of Health Stroke Scale.

Regarding functional status, total FIM score at admission was lower in patients with polypharmacy than in those without polypharmacy (68 [34-91] vs. 56 [29-82]; p = 0.048), whereas admission motor and cognitive FIM scores did not reach statistical significance. At discharge, motor, cognitive, and total FIM scores were lower in the polypharmacy group than in the non-polypharmacy group (total FIM: 107 [67-124] vs. 80 [39-113]; p < 0.001). FIM gain during hospitalization was also smaller in the polypharmacy group (27 [16-37] vs. 18 [4-32]; p < 0.001). Length of hospital stay was longer in patients with polypharmacy than in those without polypharmacy (18 [12-29] vs. 20 [14-41] days; p = 0.007). There were no statistically significant between-group differences in NIHSS at discharge or discharge destination (p = 0.092 and p = 0.084, respectively). The distribution of pre-admission medication classes according to polypharmacy status is summarized in Supplementary Table 1.

For the regression analysis, pre-stroke mRS was entered as a dichotomized variable (0-2 vs. 3-4). The results of the multiple linear regression analysis with total FIM score at discharge as the dependent variable in the whole cohort are shown in Table 2. There was no evidence of serious multicollinearity among the explanatory variables, as all VIF values were <5. After adjustment for age, sex, BMI, CCI, stroke type, pre-stroke mRS, NIHSS at admission, serum albumin at admission, and admission motor and cognitive FIM scores, pre-admission polypharmacy was independently associated with lower total FIM scores at discharge (B = −4.70, 95% confidence interval [CI] −9.07 to −0.33; p = 0.035). Visual inspection of regression diagnostics did not reveal major violations of model assumptions.

**Table 2.** Multiple Linear Regression Analysis of Factors Associated with Total FIM Score at Discharge in All Patients.

Variable	B	SE	95% CI	p Value
Age, years	−0.12	0.09	−0.29 to 0.06	0.193
Sex: female	0.90	2.08	−3.20 to 4.99	0.667
BMI, kg/m^2^	0.45	0.27	−0.08 to 0.97	0.094
Charlson Comorbidity Index	−0.36	0.70	−1.74 to 1.02	0.611
Stroke type: hemorrhagic stroke	−2.25	2.45	−7.07 to 2.58	0.360
Pre-stroke mRS: 3-4	−9.84	2.72	−15.20 to −4.49	<0.001
NIHSS at admission	−0.48	0.15	−0.77 to −0.20	0.001
Serum albumin at admission, g/dL	1.94	2.00	−1.99 to 5.87	0.332
Admission motor FIM score	0.55	0.07	0.42 to 0.68	<0.001
Admission cognitive FIM score	1.58	0.14	1.30 to 1.86	<0.001
Pre-admission polypharmacy	−4.70	2.22	−9.07 to −0.33	0.035

Multivariable linear regression analysis was performed with total FIM score at discharge as the dependent variable. All variables were entered simultaneously using the forced-entry method. B indicates the unstandardized regression coefficient. Pre-admission polypharmacy was defined as the use of five or more regular pre-admission medications. The reference categories were male sex, ischemic stroke, pre-stroke mRS 0-2, and non-polypharmacy. BMI: body mass index; CI: confidence interval; FIM: Functional Independence Measure; mRS: modified Rankin Scale; NIHSS: National Institutes of Health Stroke Scale; SE: standard error.

In the multivariable analysis for FIM gain, pre-admission polypharmacy was not independently associated with FIM gain after adjustment for baseline severity and other clinical covariates, although the estimate was in the same direction as the primary outcome analysis (B = −4.24, 95% CI −8.53 to 0.05; p = 0.053) (Table 3).

**Table 3.** Multiple Linear Regression Analysis of Factors Associated with FIM Gain in All Patients.

Variable	B	SE	95% CI	p Value
Age, years	−0.12	0.09	−0.29 to 0.05	0.173
Sex: female	0.84	2.04	−3.18 to 4.86	0.681
BMI, kg/m^2^	0.44	0.26	−0.07 to 0.96	0.093
Charlson Comorbidity Index	−0.37	0.69	−1.72 to 0.99	0.594
Stroke type: hemorrhagic stroke	−2.40	2.41	−7.14 to 2.33	0.319
Pre-stroke mRS: 3-4	−9.40	2.67	−14.66 to −4.14	0.001
NIHSS at admission	−0.47	0.14	−0.75 to −0.19	0.001
Serum albumin at admission, g/dL	1.95	1.96	−1.91 to 5.81	0.320
Admission motor FIM score	−0.46	0.06	−0.59 to −0.33	<0.001
Admission cognitive FIM score	0.62	0.14	0.34 to 0.89	<0.001
Pre-admission polypharmacy	−4.24	2.18	−8.53 to 0.05	0.053

Multiple linear regression analysis was performed with FIM gain as the dependent variable. All variables were entered simultaneously using the forced-entry method. B indicates the unstandardized regression coefficient. FIM gain was defined as discharge total FIM score minus admission total FIM score. Pre-admission polypharmacy was defined as the use of five or more regular pre-admission medications. The reference categories were male sex, ischemic stroke, pre-stroke mRS 0-2, and non-polypharmacy. BMI: body mass index; CI: confidence interval; FIM: Functional Independence Measure; mRS: modified Rankin Scale; NIHSS: National Institutes of Health Stroke Scale; SE: standard error.

Several sensitivity and supplementary analyses were performed. In the sensitivity analysis using heteroscedasticity-consistent robust standard errors, the association between pre-admission polypharmacy and lower discharge total FIM remained consistent with the primary analysis (B = −4.70, robust 95% CI −9.03 to −0.37; p = 0.033) (Supplementary Table 2). In additional sensitivity analyses including length of hospital stay as a covariate, pre-admission polypharmacy remained associated with lower discharge total FIM (B = −4.85, 95% CI −9.23 to −0.47; p = 0.030) and lower FIM gain (B = −4.39, 95% CI −8.68 to −0.09; p = 0.046) (Supplementary Table 3). However, the association with FIM gain after additional adjustment for length of hospital stay should be interpreted cautiously because the magnitude of the association was modest.

To examine the robustness of the association in older patients, we performed an additional analysis restricted to patients aged ≥65 years (n = 235) (Table 4). In this analysis, pre-admission polypharmacy remained associated with lower total FIM scores at discharge (B = −5.25, 95% CI −10.16 to −0.33; P = 0.037).

**Table 4.** Multiple Linear Regression Analysis of Factors Associated with Total FIM Score at Discharge in Patients Aged ≥65 Years.

Variable	B	SE	95% CI	p Value
Age, years	−0.03	0.16	−0.34 to 0.28	0.850
Sex: female	−2.02	2.35	−6.66 to 2.61	0.391
BMI, kg/m^2^	0.41	0.33	−0.24 to 1.06	0.213
Charlson Comorbidity Index	−0.42	0.78	−1.96 to 1.13	0.596
Stroke type: hemorrhagic stroke	−4.38	3.03	−10.36 to 1.60	0.150
Pre-stroke mRS: 3-4	−7.45	2.89	−13.14 to −1.76	0.011
NIHSS at admission	−0.37	0.16	−0.68 to −0.05	0.022
Serum albumin at admission, g/dL	2.77	2.47	−2.11 to 7.65	0.265
Admission motor FIM score	0.68	0.08	0.53 to 0.83	<0.001
Admission cognitive FIM score	1.57	0.16	1.26 to 1.88	<0.001
Pre-admission polypharmacy	−5.25	2.49	−10.16 to −0.33	0.037

Multivariable linear regression analysis was performed with total FIM score at discharge as the dependent variable in patients aged ≥65 years. The same covariates as in Table 2 were included in the model. All variables were entered simultaneously using the forced-entry method. B indicates the unstandardized regression coefficient. Pre-admission polypharmacy was defined as the use of five or more regular pre-admission medications. The reference categories were male sex, ischemic stroke, pre-stroke mRS 0-2, and non-polypharmacy. BMI: body mass index; CI: confidence interval; FIM: Functional Independence Measure; mRS: modified Rankin Scale; NIHSS: National Institutes of Health Stroke Scale; SE: standard error.

Supplementary age-stratified analyses are shown in Supplementary Table 4. A statistically significant association between pre-admission polypharmacy and lower discharge total FIM was observed in the 65-74-year age group, whereas no statistically significant association was observed in the 18-64-year or ≥75-year groups. However, the interaction between pre-admission polypharmacy and age group was not statistically significant (p for interaction = 0.182).

In an exploratory multivariable linear regression analysis additionally adjusted for psychotropic drug use, the association between pre-admission polypharmacy and lower discharge total FIM was attenuated and no longer reached statistical significance (B = −4.07, 95% CI −8.50 to 0.35; p = 0.071) (Supplementary Table 5). Psychotropic drug use itself was not independently associated with discharge total FIM (B = −4.81, 95% CI −10.66 to 1.04; p = 0.107).

In a supplementary analysis using the number of pre-admission medications as a continuous variable instead of binary polypharmacy, a greater medication count was associated with lower discharge total FIM after adjustment for the same covariates used in the primary model (B = −0.55, 95% CI −1.08 to −0.03; p = 0.040) (Supplementary Table 6).

## Discussion

In this retrospective observational study of patients with acute stroke, pre-admission polypharmacy was associated with lower total FIM scores at discharge after adjustment for age, sex, BMI, CCI, stroke type, pre-stroke mRS, NIHSS at admission, serum albumin at admission, and admission motor and cognitive FIM scores. For discharge total FIM, this association remained consistent in sensitivity analyses using heteroscedasticity-consistent robust standard errors, after additional adjustment for length of hospital stay, and in the analysis restricted to patients aged ≥65 years. In contrast, pre-admission polypharmacy was not independently associated with FIM gain in the multivariable model for the secondary outcome, although the estimate was in the same direction as that observed for discharge total FIM. These findings suggest that pre-admission polypharmacy may be more appropriately interpreted as a marker of lower functional status at discharge rather than as direct evidence of impaired functional recovery during hospitalization.

The findings of this study are broadly consistent with previous reports showing associations between polypharmacy and poor health-related outcomes ^(1), (2)^. In community-dwelling and hospitalized older adults, a greater number of prescribed medications has been associated with falls ^(5)^, decline in physical function ^(4)^, prolonged length of hospital stay ^(20)^, and mortality ^(7), (8)^. In stroke populations, polypharmacy and potentially inappropriate medication use have also been associated with poorer ADL and lower rates of discharge to home ^(21)^. Consistent with these studies, patients with pre-admission polypharmacy in the present cohort had lower functional status at discharge than those without polypharmacy. To our knowledge, few studies have examined whether pre-admission polypharmacy is associated with ADL status at discharge in patients with acute stroke after accounting for pre-stroke disability, stroke severity, and admission motor and cognitive FIM scores. However, because the association with FIM gain was not statistically significant after adjustment for baseline severity and functional status, the present findings should not be interpreted as showing that polypharmacy directly suppresses functional recovery.

One possible interpretation is that pre-admission polypharmacy reflects underlying clinical complexity ^(4), (22)^. In the present study, patients with polypharmacy were older and had a greater comorbidity burden, worse pre-stroke mRS, and lower total FIM scores at admission. Polypharmacy may therefore represent a clinical marker of multimorbidity, frailty, cognitive and physical vulnerability, or other factors that are difficult to fully capture in routinely collected clinical data ^(4), (22)^. Although we adjusted for major prognostic factors, residual confounding by unmeasured clinical complexity remains likely. Variables, such as detailed frailty status, premorbid cognitive function, delirium during hospitalization, rehabilitation dose and intensity, in-hospital complications, medication appropriateness, and social support were not available in this retrospective dataset. Therefore, the observed association should be interpreted as an association rather than a causal effect of medication burden itself.

Medication-related mechanisms may also partly contribute to the observed association. Certain medication classes, including hypnotics, anxiolytics, antipsychotics, analgesics, and strong anticholinergic drugs, may plausibly affect attention, alertness, or balance, thereby limiting participation in rehabilitation ^(17), (18), (23)^. To improve transparency, we added supplementary information on pre-admission medication classes and strong anticholinergic drug use. In the exploratory model additionally adjusted for psychotropic drug use, the association between pre-admission polypharmacy and discharge total FIM was attenuated and no longer reached statistical significance, whereas psychotropic drug use itself was not independently associated with discharge total FIM. This finding may suggest partial overlap between psychotropic drug use and the overall medication burden captured by polypharmacy. However, because this analysis was exploratory and the number of patients using psychotropic drugs was limited, the lack of statistical significance should not be interpreted as evidence that psychotropic drug use has no clinical relevance.

The clinical magnitude of the association also requires cautious interpretation. Although pre-admission polypharmacy was statistically associated with lower discharge total FIM, the adjusted difference was smaller than the 22-point MCID used in the sample size calculation. Therefore, the clinical significance of this difference at the individual patient level may be limited. In addition, length of hospital stay was not included in the primary model because it may lie on the causal pathway between medication burden and discharge functional status. The association with FIM gain reached statistical significance only in the sensitivity analysis additionally adjusted for length of hospital stay; however, this finding should be interpreted cautiously because adjustment for length of stay may introduce overadjustment. Taken together, these results support interpreting pre-admission polypharmacy primarily as a marker for risk stratification rather than as a confirmed modifiable determinant of functional recovery.

From a clinical perspective, pre-admission polypharmacy or a greater number of pre-admission medications may be useful as an early and easily identifiable marker for patients at risk of lower functional status at discharge. Patients with polypharmacy may require closer multidisciplinary assessment, rehabilitation planning, and discharge preparation from the early phase of hospitalization. Medication review may also be clinically reasonable as part of comprehensive stroke care. However, the present study did not evaluate the effects of deprescribing, medication optimization, or specific medication classes on functional outcomes. Future prospective studies are needed to determine whether structured medication review or deprescribing interventions can improve rehabilitation-related outcomes after stroke.

This study has several limitations. First, because of its retrospective observational design at a single center, causal inferences cannot be made, and the generalizability of the findings may be limited. Second, although we adjusted for major measured prognostic factors, residual confounding by unmeasured clinical complexity cannot be ruled out. Third, the study was designed primarily to examine overall medication burden and was not intended to determine the independent effects of specific drug classes or pharmacologic burden indices, such as anticholinergic burden, sedative load, or potentially inappropriate medication use. Fourth, patients who died during hospitalization were excluded from the analysis. If medication burden and clinical severity were both associated with in-hospital mortality and discharge functional status among survivors, excluding patients who died may have biased the estimated association. Therefore, our findings should be generalized with caution and primarily apply to patients who survived hospitalization and had complete outcome data.

Despite these limitations, this study has several strengths. It focused on patients with acute stroke and evaluated FIM at both admission and discharge, capturing both motor and cognitive aspects of ADL. Major prognostic factors, including age, comorbidity burden, pre-stroke functional status, stroke severity, and baseline FIM, were simultaneously adjusted for. In addition, several sensitivity and supplementary analyses were conducted, including robust standard error estimation, adjustment for length of hospital stay, analysis restricted to older patients, psychotropic drug-adjusted analysis, and analysis using medication count as a continuous variable. These analyses helped assess the robustness of the findings.

### Conclusion

Pre-admission polypharmacy was associated with lower total FIM scores at discharge, but not with FIM gain, in patients with acute stroke after adjustment for major clinical covariates. These findings suggest that pre-admission polypharmacy may serve as a marker of clinical complexity and help identify patients at risk of poorer functional status at discharge. Further prospective studies are needed to clarify whether medication burden is causally related to functional outcomes and whether medication-focused interventions provide clinical benefit in this population.

## Article Information

### Acknowledgments

The author would like to thank the physicians, nurses, rehabilitation staff, ward pharmacists, and patients who contributed to this study. The author particularly acknowledges the ward pharmacists for their important role in medication reconciliation and medication-related data collection. The author also thanks Ivi Vaher for assistance with English language editing and for reviewing the manuscript for clarity, consistency, and reporting transparency.

### Conflicts of Interest

None

### Institutional Review Board Approval Code and Name of the Institution

The study protocol was approved by the Ethics Committee of Kyorin University School of Medicine (approval no. R04-045). Informed consent was waived because of the retrospective observational design. Instead, an opt-out consent procedure was implemented by disclosing the study information on the institutional website and allowing patients to refuse participation.

## Supplement

Supplementary Material
