# Supplementary material for: Association between Pre-Admission Polypharmacy and Functional Status at Discharge in Patients with Acute Stroke: A Retrospective Observational Study
Source: JMA J. 2026 Jul 10;9(5):1244–53. doi: 10.31662/jmaj.2025-0597 (PMC13639490; doi:10.31662/jmaj.2025-0597)
Supplement: Supplementary file 1 [file 2433-3298-9-5-1244-s001.pdf]

Supplementary Table 1. Pre-admission medication classes in the overall cohort and according to polypharmacy status

Medication class	Overall, n (%)	Non-polypharmacy, n (%)	Polypharmacy, n (%)	P value
Antithrombotic agents	94 (31.0)	15 (11.3)	79 (46.5)	<0.001
Antihypertensive agents	143 (47.2)	26 (19.5)	117 (68.8)	<0.001
Lipid-lowering agents	77 (25.4)	11 (8.3)	66 (38.8)	<0.001
Antidiabetic agents	55 (18.2)	6 (4.5)	49 (28.8)	<0.001
Gastric acid suppressants	69 (22.8)	7 (5.3)	62 (36.5)	<0.001
Psychotropic drugs*	39 (12.9)	7 (5.3)	32 (18.8)	0.001
Hypnotics	29 (9.6)	4 (3.0)	25 (14.7)	<0.001
Anxiolytics	9 (3.0)	1 (0.8)	8 (4.7)	0.083
Antidepressants	4 (1.3)	1 (0.8)	3 (1.8)	0.634
Antipsychotics	4 (1.3)	1 (0.8)	3 (1.8)	0.634
Analgesics†	33 (10.9)	4 (3.0)	29 (17.1)	<0.001
Antiepileptic drugs	7 (2.3)	0 (0.0)	7 (4.1)	0.019
Strong anticholinergic drugs‡	6 (2.0)	1 (0.8)	5 (2.9)	0.235

Data are presented as n (%). P values were calculated using the chi-square test or Fisher’s exact test, as appropriate. Pre-admission polypharmacy was defined as the use of five or more regular pre-admission medications.

*Psychotropic drugs were defined as hypnotics, anxiolytics, antidepressants, or antipsychotics.

†Analgesics were recorded as a separate medication class and were not included in psychotropic drugs.

‡Strong anticholinergic drugs were defined as medications with an Anticholinergic Cognitive Burden score of 3; patients were classified as users if they used at least one such medication.

Supplementary Table 2. Robust standard error sensitivity analysis for the association between pre-admission polypharmacy and discharge total FIM

Outcome	Variable	B	Robust SE	Robust 95% CI	P value
Discharge total FIM	Pre-admission polypharmacy	−4.70	2.20	−9.03 to −0.37	0.033

Robust standard errors were estimated using heteroscedasticity-consistent covariance matrix estimation with the HC3 estimator. The same covariates as in the primary model shown in Table 2 were included. B indicates the unstandardized regression coefficient. Pre-admission polypharmacy was defined as the use of five or more regular pre-admission medications.

Abbreviations: CI, confidence interval; FIM, Functional Independence Measure; SE, standard error.

Supplementary Table 3. Sensitivity analyses additionally adjusted for length of hospital stay

Outcome	Variable	B	SE	95% CI	P value
Discharge total FIM	Pre-admission polypharmacy	−4.85	2.23	−9.23 to −0.47	0.030
FIM gain	Pre-admission polypharmacy	−4.39	2.18	−8.68 to −0.09	0.046

Additional sensitivity analyses were performed by adding length of hospital stay to the covariates used in the corresponding primary models. For discharge total FIM, the same covariates as in Table 2 were included. For FIM gain, the same covariates as in Table 3 were included. B indicates the unstandardized regression coefficient. Pre-admission polypharmacy was defined as the use of five or more regular pre-admission medications.

Abbreviations: CI, confidence interval; FIM, Functional Independence Measure; SE, standard error.

Supplementary Table 4. Age-stratified multivariable linear regression analyses of the association between pre-admission polypharmacy and discharge total FIM

Age stratum	n	B for pre-admission polypharmacy	SE	95% CI	P value
18–64 years	68	−2.64	4.23	−11.10 to 5.82	0.535
65–74 years	62	−12.46	4.33	−21.13 to −3.79	0.006
≥75 years	173	−1.36	2.84	−6.97 to 4.25	0.633

B values represent unstandardized regression coefficients for pre-admission polypharmacy. Analyses were performed using a parsimonious multivariable linear regression model including pre-admission polypharmacy, age, pre-stroke mRS, NIHSS at admission, and admission motor and cognitive FIM scores. The interaction between pre-admission polypharmacy and age group was not statistically significant (P for interaction = 0.182).

Abbreviations: CI, confidence interval; FIM, Functional Independence Measure; mRS, modified Rankin Scale; NIHSS, National Institutes of Health Stroke Scale; SE, standard error.

Supplementary Table 5. Multivariable linear regression analysis additionally adjusted for psychotropic drug use

Variable	B	SE	95% CI	P value
Age, years	−0.12	0.09	−0.30 to 0.05	0.177
Sex: female	1.34	2.09	−2.78 to 5.45	0.524
BMI, kg/m²	0.41	0.27	−0.11 to 0.94	0.124
Charlson Comorbidity Index	−0.36	0.70	−1.74 to 1.02	0.607
Stroke type: hemorrhagic stroke	−2.00	2.45	−6.82 to 2.82	0.416
Pre-stroke mRS: 3–4	−9.42	2.73	−14.79 to −4.06	0.001
NIHSS at admission	−0.49	0.15	−0.78 to −0.20	0.001
Serum albumin at admission, g/dL	1.92	1.99	−1.99 to 5.84	0.334
Admission motor FIM score	0.54	0.07	0.41 to 0.67	<0.001
Admission cognitive FIM score	1.60	0.14	1.32 to 1.87	<0.001
Pre-admission polypharmacy	−4.07	2.25	−8.50 to 0.35	0.071
Psychotropic drug use	−4.81	2.97	−10.66 to 1.04	0.107

Multiple linear regression analysis was performed with total FIM score at discharge as the dependent variable. All variables were entered simultaneously into the model. B indicates the unstandardized regression coefficient. Pre-admission polypharmacy was defined as the use of five or more regular pre-admission medications. Psychotropic drug use was defined as the use of hypnotics, anxiolytics, antidepressants, or antipsychotics. The reference categories were male sex, ischemic stroke, pre-stroke mRS 0–2, non-polypharmacy, and no psychotropic drug use.

Abbreviations: BMI, body mass index; CI, confidence interval; FIM, Functional Independence Measure; mRS, modified Rankin Scale; NIHSS, National Institutes of Health Stroke Scale; SE, standard error.

Supplementary Table 6. Multivariable linear regression analysis of the association between medication count and discharge total FIM

Variable	B	SE	95% CI	P value
Age, years	−0.13	0.09	−0.30 to 0.04	0.134
Sex: female	1.22	2.08	−2.88 to 5.32	0.558
BMI, kg/m²	0.42	0.27	−0.11 to 0.94	0.118
Charlson Comorbidity Index	−0.36	0.70	−1.74 to 1.03	0.613
Stroke type: hemorrhagic stroke	−2.08	2.45	−6.90 to 2.75	0.398
Pre-stroke mRS: 3–4	−9.81	2.73	−15.17 to −4.44	<0.001
NIHSS at admission	−0.48	0.15	−0.77 to −0.19	0.001
Serum albumin at admission, g/dL	1.73	2.00	−2.19 to 5.66	0.385
Admission motor FIM score	0.54	0.07	0.41 to 0.67	<0.001
Admission cognitive FIM score	1.59	0.14	1.31 to 1.87	<0.001
Number of pre-admission medications	−0.55	0.27	−1.08 to −0.03	0.040

Multiple linear regression analysis was performed with total FIM score at discharge as the dependent variable. All variables were entered simultaneously using the forced-entry method. B indicates the unstandardized regression coefficient. Medication count was defined as the number of regular pre-admission medications. The reference categories were male sex, ischemic stroke, and pre-stroke mRS 0–2.

Abbreviations: BMI, body mass index; CI, confidence interval; FIM, Functional Independence Measure; mRS, modified Rankin Scale; NIHSS, National Institutes of Health Stroke Scale; SE, standard error.
